# Diethyl 2-[(3,5-dimethyl-1*H*-pyrazol-1-yl)(4-meth­oxy­phen­yl)meth­yl]propane­dioate

**DOI:** 10.1107/S1600536810025572

**Published:** 2010-07-10

**Authors:** Ihssan Meskini, Maria Daoudi, Jean-Claude Daran, Abdelali Kerbal, Hafid Zouihri

**Affiliations:** a Laboratoire de Chimie Organique, Faculté des Sciences Dhar el Mahraz, Université Sidi Mohammed Ben Abdellah, Fès, Morocco; b Laboratoire de Chimie de Coordination, 205 Route de Narbonne, 31077 Toulouse Cedex, France; c Laboratoires de Diffraction des Rayons X, Division UATRS, Centre National pour la Recherche Scientifique et Technique, Rabat, Morocco

## Abstract

The title compound, C_20_H_26_N_2_O_5_, was prepared in good yield (76%) through condensation of diethyl (4-meth­oxy­benz­yl)propane­dioate with 3,5-dimethyl-1*H*-pyrazole. The dihedral between the benzene and pyrazole rings is 83.96 (10)°. The crystal packing is stabilized by a C—H⋯O inter­action, which links the mol­ecules into centrosymmetric dimers.

## Related literature

For related compounds displaying biological activity, see: Dayam *et al.* (2007 ▶); Patil *et al.* (2007 ▶); Ramkumar *et al.* (2008 ▶); Sechi *et al.* (2009 ▶) & Zeng *et al.* (2008 ▶). For the synthetic procedure, see: Pommier & Neamati (2006 ▶).
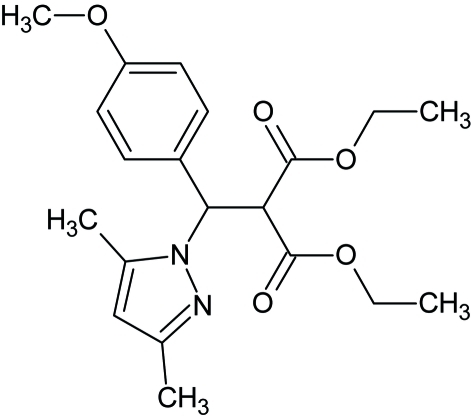


## Experimental

### 

#### Crystal data

C_20_H_26_N_2_O_5_
*M* *_r_* = 374.43
Monoclinic, 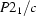

*a* = 11.9618 (3) Å
*b* = 7.9681 (2) Å
*c* = 21.1269 (6) Å
β = 96.504 (1)°
*V* = 2000.70 (9) Å^3^
*Z* = 4
Mo *K*α radiation
μ = 0.09 mm^−1^
*T* = 296 K
0.23 × 0.17 × 0.14 mm

#### Data collection

Bruker X8 APEXII CCD area-detector diffractometer
18616 measured reflections
3921 independent reflections
3177 reflections with *I* > 2σ(*I*)
*R*_int_ = 0.027

#### Refinement

*R*[*F* ^2^ > 2σ(*F* ^2^)] = 0.056
*wR*(*F* ^2^) = 0.154
*S* = 1.05
3921 reflections
249 parameters
H-atom parameters constrained
Δρ_max_ = 0.68 e Å^−3^
Δρ_min_ = −0.45 e Å^−3^

### 

Data collection: *APEX2* (Bruker, 2005 ▶); cell refinement: *SAINT* (Bruker, 2005 ▶); data reduction: *SAINT*; program(s) used to solve structure: *SHELXS97* (Sheldrick, 2008 ▶); program(s) used to refine structure: *SHELXL97* (Sheldrick, 2008 ▶); molecular graphics: *PLATON* (Spek, 2009 ▶); software used to prepare material for publication: *publCIF* (Westrip, 2010 ▶).

## Supplementary Material

Crystal structure: contains datablocks I, global. DOI: 10.1107/S1600536810025572/bt5280sup1.cif

Structure factors: contains datablocks I. DOI: 10.1107/S1600536810025572/bt5280Isup2.hkl

Additional supplementary materials:  crystallographic information; 3D view; checkCIF report

**Fig. 3.**
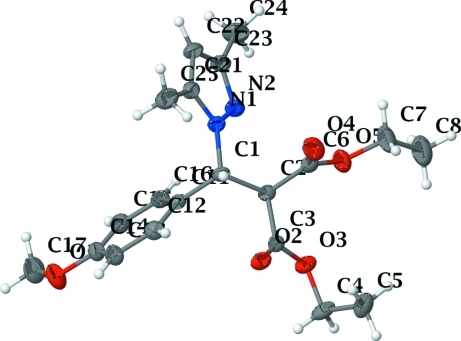
View of the title compound showing displacement ellipsoids at the 50% probability level.

Enhanced figure: interactive version of Fig. 3

## Figures and Tables

**Table 1:** Hydrogen-bond geometry (Å, °)

*D*—H⋯*A*	*D*—H	H⋯*A*	*D*⋯*A*	*D*—H⋯*A*
C12—H12⋯O2^i^	0.93	2.51	3.358 (3)	152

Symmetry code: (i) 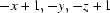.

**Fig. 1.**
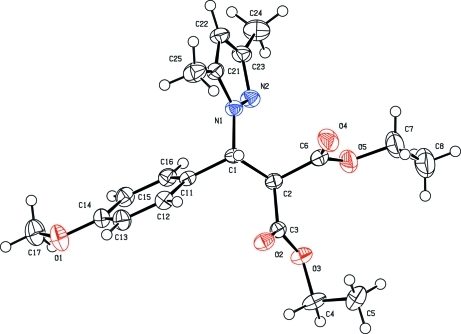
Molecular structure of the title compound with the atom-labelling scheme. Displacement ellipsoids are drawn at the 30% probability level. H atoms are represented as small spheres of arbitrary radii.

**Fig. 2.**
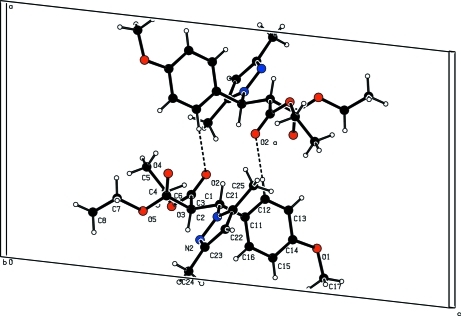
Partial packing view showing the chain generated by C—H···O hydrogen bonds shown as dashed lines. Symmetry code for generating the second molecule: 1 - x,-y,1 - z.

## supplementary crystallographic information

### Comment

For the rational design of new HIV-1 Integrase (H—I) inhibitors, one validated target for chemotherapeutic intervention (Dayam *et al., *2007), is fundamentally based on intermolecular coordination between H—I / chemical inhibitor / metals (Mg^+2^ and Mn^+2^, co-factors of the enzyme), leading to the formation of bimetallic complexes (Zeng *et al., *2008; Sechi *et al.*, 2009). Thereby, several bimetallic metal complexes, in many cases exploring the known-well polydentate ligands, appear in this scenario as the most promising concept to be employed in either enzyme / drug interaction or electron transfer process, in the last case involving the biological oxygen transfer (Sechi *et al., *2009; Ramkumar *et al., *2008). Another exciting example of application for such polydentate ligands involves the synergic water activation, that occurs *via* the so-called -remote metallic atoms. Such organometallic compounds are structurally deemed to promote or block the H—I activity (Zeng *et al., *2008).

In the molecule of the title compound (Fig.1), the dihedral angle between the planes of the pheny and the pyrazol ring is 83.96 (10)°.

### Experimental

To a solution of the diethyl (4-methoxybenzyl)propanedioate (5 mmol) in water (20 ml) was added the 3,5-dimethyl-1*H*-pyrazole (6 mmol) and the mixture and the stirring was continued at room temperature until the complete consume of the starting material. After removing solvent, the crude products were dissolved in diethyl ether (2x40 ml) and washed with water until the pH became neutral. The organic solvent was dried with sodium sulfate and then evaporated to give the pure compound (I) with 76% yield.. White crystals are obtained by recrystallization in ether/hexane (2/1).

Suitable single-crystal of malonate derivative (I) was obtained by recrystallization from ethanol. A white-transparent crystal was mounted on a glass fibre.

### Refinement

All H atoms attached to C atoms were fixed geometrically and treated as riding with C—H = 0.96 Å (methyl), C—H = 0.93 Å (aromatic), 0.97 Å (methylene) and 0.98 Å (methine) with *U*_iso_(H) = 1.2*U*_eq_ or *U*_iso_(H) = 1.5*U*_eq_(methyl).

### Crystal data

**Table:**

C_20_H_26_N_2_O_5_	*F*(000) = 800
*M**_r_* = 374.43	*D*_x_ = 1.243 Mg m^−^^3^
Monoclinic, *P*2_1_/*c*	Melting point: 361 K
Hall symbol: -P 2ybc	Mo *K*α radiation, λ = 0.71073 Å
*a* = 11.9618 (3) Å	Cell parameters from 2174 reflections
*b* = 7.9681 (2) Å	θ = 2.3–27.1°
*c* = 21.1269 (6) Å	µ = 0.09 mm^−^^1^
β = 96.504 (1)°	*T* = 296 K
*V* = 2000.70 (9) Å^3^	Block, colourless
*Z* = 4	0.23 × 0.17 × 0.14 mm

### Data collection

**Table:**

Bruker X8 APEXII CCD area-detector diffractometer	3177 reflections with *I* > 2σ(*I*)
Radiation source: fine-focus sealed tube	*R*_int_ = 0.027
graphite	θ_max_ = 26.0°, θ_min_ = 2.7°
φ and ω scans	*h* = −14→14
18616 measured reflections	*k* = −9→9
3921 independent reflections	*l* = −26→26

### Refinement

**Table:**

Refinement on *F*^2^	Primary atom site location: structure-invariant direct methods
Least-squares matrix: full	Secondary atom site location: difference Fourier map
*R*[*F*^2^ > 2σ(*F*^2^)] = 0.056	Hydrogen site location: inferred from neighbouring sites
*wR*(*F*^2^) = 0.154	H-atom parameters constrained
*S* = 1.05	*w* = 1/[σ^2^(*F*_o_^2^) + (0.0687*P*)^2^ + 1.8319*P*] where *P* = (*F*_o_^2^ + 2*F*_c_^2^)/3
3921 reflections	(Δ/σ)_max_ = 0.007
249 parameters	Δρ_max_ = 0.68 e Å^−^^3^
0 restraints	Δρ_min_ = −0.45 e Å^−^^3^

### Special details

**Table:**

Experimental. The data collection nominally covered a sphere of reciprocal space, by a combination of tree sets of exposures; each set had a different φ angle for the crystal and each exposure covered 0.5° in ω and 20 s in time. The crystal-to-detector distance was 37.5 mm.
Geometry. All e.s.d.'s (except the e.s.d. in the dihedral angle between two l.s. planes) are estimated using the full covariance matrix. The cell e.s.d.'s are taken into account individually in the estimation of e.s.d.'s in distances, angles and torsion angles; correlations between e.s.d.'s in cell parameters are only used when they are defined by crystal symmetry. An approximate (isotropic) treatment of cell e.s.d.'s is used for estimating e.s.d.'s involving l.s. planes.
Refinement. Refinement of *F*^2^ against ALL reflections. The weighted *R*-factor *wR* and goodness of fit *S* are based on *F*^2^, conventional *R*-factors *R* are based on *F*, with *F* set to zero for negative *F*^2^. The threshold expression of *F*^2^ > σ(*F*^2^) is used only for calculating *R*-factors(gt) *etc*. and is not relevant to the choice of reflections for refinement. *R*-factors based on *F*^2^ are statistically about twice as large as those based on *F*, and *R*- factors based on ALL data will be even larger.

### Fractional atomic coordinates and isotropic or equivalent isotropic displacement parameters (Å^2^)

**Table:**

	*x*	*y*	*z*	*U*_iso_*/*U*_eq_	
O2	0.40914 (11)	−0.14261 (19)	0.44322 (7)	0.0301 (3)	
O3	0.26937 (12)	−0.19794 (18)	0.36564 (7)	0.0321 (4)	
O4	0.39768 (14)	0.2442 (2)	0.36381 (8)	0.0441 (4)	
O5	0.23951 (15)	0.1580 (2)	0.30637 (7)	0.0446 (4)	
O1	0.16987 (15)	−0.2065 (2)	0.68944 (8)	0.0468 (5)	
N1	0.25069 (14)	0.3219 (2)	0.47437 (8)	0.0252 (4)	
N2	0.15274 (14)	0.3525 (2)	0.43620 (8)	0.0297 (4)	
C1	0.29918 (16)	0.1528 (2)	0.47752 (9)	0.0230 (4)	
H1	0.3813	0.1633	0.4845	0.028*	
C2	0.26772 (16)	0.0673 (3)	0.41296 (9)	0.0244 (4)	
H2	0.1859	0.0541	0.4051	0.029*	
C11	0.26097 (16)	0.0542 (2)	0.53261 (9)	0.0229 (4)	
C3	0.32439 (16)	−0.1032 (2)	0.41081 (9)	0.0239 (4)	
C12	0.33950 (17)	−0.0251 (3)	0.57616 (9)	0.0277 (4)	
H12	0.4154	−0.0197	0.5706	0.033*	
C21	0.28468 (17)	0.4563 (3)	0.51094 (10)	0.0285 (5)	
C16	0.14790 (16)	0.0419 (3)	0.54174 (10)	0.0277 (4)	
H16	0.0941	0.0929	0.5127	0.033*	
C13	0.30680 (18)	−0.1116 (3)	0.62736 (10)	0.0323 (5)	
H13	0.3605	−0.1643	0.6559	0.039*	
C14	0.19347 (18)	−0.1206 (3)	0.63658 (10)	0.0311 (5)	
C15	0.11364 (17)	−0.0445 (3)	0.59307 (10)	0.0312 (5)	
H15	0.0376	−0.0515	0.5983	0.037*	
C6	0.30985 (19)	0.1697 (3)	0.35912 (10)	0.0308 (5)	
C23	0.12710 (18)	0.5105 (3)	0.44907 (11)	0.0322 (5)	
C4	0.32363 (19)	−0.3556 (3)	0.35159 (12)	0.0370 (5)	
H4A	0.2679	−0.4329	0.3314	0.044*	
H4B	0.3575	−0.4063	0.3909	0.044*	
C22	0.20634 (18)	0.5797 (3)	0.49546 (10)	0.0330 (5)	
H22	0.2060	0.6874	0.5124	0.040*	
C5	0.4121 (2)	−0.3243 (4)	0.30837 (11)	0.0466 (6)	
H5A	0.3789	−0.2693	0.2704	0.070*	
H5B	0.4442	−0.4292	0.2973	0.070*	
H5C	0.4699	−0.2542	0.3296	0.070*	
C25	0.3888 (2)	0.4563 (3)	0.55689 (12)	0.0416 (6)	
H25A	0.3743	0.4000	0.5953	0.062*	
H25B	0.4114	0.5699	0.5666	0.062*	
H25C	0.4478	0.3990	0.5384	0.062*	
C7	0.2781 (3)	0.2351 (4)	0.24975 (12)	0.0612 (8)	
H7A	0.2695	0.3560	0.2515	0.073*	
H7B	0.3571	0.2098	0.2481	0.073*	
C24	0.0258 (2)	0.5924 (3)	0.41410 (14)	0.0489 (7)	
H24A	0.0493	0.6722	0.3842	0.073*	
H24B	−0.0160	0.6490	0.4439	0.073*	
H24C	−0.0209	0.5086	0.3917	0.073*	
C17	0.0566 (2)	−0.2040 (4)	0.70479 (13)	0.0545 (7)	
H17A	0.0335	−0.0900	0.7101	0.082*	
H17B	0.0521	−0.2649	0.7436	0.082*	
H17C	0.0081	−0.2554	0.6709	0.082*	
C8	0.2127 (3)	0.1700 (5)	0.19471 (13)	0.0731 (10)	
H8A	0.2245	0.0511	0.1922	0.110*	
H8B	0.2351	0.2230	0.1573	0.110*	
H8C	0.1345	0.1919	0.1975	0.110*	

### Atomic displacement parameters (Å^2^)

**Table:**

	*U*^11^	*U*^22^	*U*^33^	*U*^12^	*U*^13^	*U*^23^
O2	0.0249 (7)	0.0292 (8)	0.0357 (8)	0.0049 (6)	0.0007 (6)	−0.0048 (6)
O3	0.0317 (8)	0.0265 (8)	0.0371 (8)	0.0009 (6)	−0.0005 (6)	−0.0089 (6)
O4	0.0466 (10)	0.0436 (10)	0.0435 (9)	−0.0125 (8)	0.0117 (8)	0.0046 (8)
O5	0.0624 (11)	0.0441 (10)	0.0264 (8)	−0.0085 (8)	0.0010 (7)	0.0036 (7)
O1	0.0475 (10)	0.0586 (12)	0.0360 (9)	−0.0031 (9)	0.0117 (7)	0.0143 (8)
N1	0.0258 (8)	0.0229 (9)	0.0265 (8)	0.0027 (7)	0.0013 (7)	−0.0005 (7)
N2	0.0280 (9)	0.0278 (9)	0.0330 (9)	0.0061 (7)	0.0015 (7)	0.0008 (7)
C1	0.0216 (9)	0.0196 (9)	0.0277 (10)	0.0025 (7)	0.0022 (7)	−0.0011 (8)
C2	0.0221 (9)	0.0238 (10)	0.0273 (10)	0.0019 (8)	0.0031 (8)	−0.0012 (8)
C11	0.0242 (9)	0.0203 (10)	0.0246 (9)	0.0011 (8)	0.0036 (7)	−0.0037 (7)
C3	0.0230 (10)	0.0237 (10)	0.0257 (9)	−0.0028 (8)	0.0067 (8)	−0.0018 (8)
C12	0.0228 (10)	0.0311 (11)	0.0287 (10)	0.0005 (8)	0.0009 (8)	−0.0014 (8)
C21	0.0320 (11)	0.0253 (11)	0.0294 (10)	−0.0012 (8)	0.0088 (8)	−0.0025 (8)
C16	0.0242 (10)	0.0293 (11)	0.0292 (10)	0.0060 (8)	0.0014 (8)	−0.0008 (8)
C13	0.0315 (11)	0.0372 (13)	0.0268 (10)	0.0019 (9)	−0.0022 (8)	0.0033 (9)
C14	0.0379 (12)	0.0310 (12)	0.0253 (10)	−0.0017 (9)	0.0073 (9)	0.0011 (8)
C15	0.0255 (10)	0.0350 (12)	0.0340 (11)	0.0006 (9)	0.0078 (8)	−0.0019 (9)
C6	0.0405 (12)	0.0244 (11)	0.0277 (10)	0.0041 (9)	0.0041 (9)	−0.0028 (8)
C23	0.0340 (11)	0.0253 (11)	0.0385 (11)	0.0075 (9)	0.0100 (9)	0.0030 (9)
C4	0.0369 (12)	0.0279 (11)	0.0454 (13)	0.0010 (9)	0.0015 (10)	−0.0159 (10)
C22	0.0401 (12)	0.0222 (10)	0.0384 (12)	0.0037 (9)	0.0122 (10)	−0.0033 (9)
C5	0.0504 (15)	0.0548 (16)	0.0350 (12)	0.0052 (12)	0.0069 (11)	−0.0127 (12)
C25	0.0425 (13)	0.0381 (13)	0.0423 (13)	−0.0001 (11)	−0.0034 (10)	−0.0091 (11)
C7	0.097 (2)	0.0572 (18)	0.0293 (13)	−0.0235 (17)	0.0078 (14)	0.0054 (12)
C24	0.0432 (14)	0.0413 (15)	0.0613 (16)	0.0180 (11)	0.0019 (12)	0.0046 (13)
C17	0.0596 (17)	0.0595 (18)	0.0492 (15)	−0.0086 (14)	0.0274 (13)	0.0059 (13)
C8	0.113 (3)	0.072 (2)	0.0346 (14)	−0.033 (2)	0.0104 (16)	−0.0004 (14)

### Geometric parameters (Å, °)

**Table:**

O2—C3	1.199 (2)	C13—H13	0.9300
O3—C3	1.331 (2)	C14—C15	1.388 (3)
O3—C4	1.460 (3)	C15—H15	0.9300
O4—C6	1.201 (3)	C23—C22	1.397 (3)
O5—C6	1.322 (3)	C23—C24	1.496 (3)
O5—C7	1.465 (3)	C4—C5	1.495 (3)
O1—C14	1.367 (3)	C4—H4A	0.9700
O1—C17	1.428 (3)	C4—H4B	0.9700
N1—C21	1.355 (3)	C22—H22	0.9300
N1—N2	1.367 (2)	C5—H5A	0.9600
N1—C1	1.466 (2)	C5—H5B	0.9600
N2—C23	1.331 (3)	C5—H5C	0.9600
C1—C11	1.517 (3)	C25—H25A	0.9600
C1—C2	1.533 (3)	C25—H25B	0.9600
C1—H1	0.9800	C25—H25C	0.9600
C2—C3	1.521 (3)	C7—C8	1.424 (4)
C2—C6	1.531 (3)	C7—H7A	0.9700
C2—H2	0.9800	C7—H7B	0.9700
C11—C12	1.390 (3)	C24—H24A	0.9600
C11—C16	1.391 (3)	C24—H24B	0.9600
C12—C13	1.376 (3)	C24—H24C	0.9600
C12—H12	0.9300	C17—H17A	0.9600
C21—C22	1.372 (3)	C17—H17B	0.9600
C21—C25	1.490 (3)	C17—H17C	0.9600
C16—C15	1.385 (3)	C8—H8A	0.9600
C16—H16	0.9300	C8—H8B	0.9600
C13—C14	1.393 (3)	C8—H8C	0.9600
			
C3—O3—C4	116.03 (16)	N2—C23—C24	120.3 (2)
C6—O5—C7	115.4 (2)	C22—C23—C24	128.4 (2)
C14—O1—C17	117.87 (19)	O3—C4—C5	110.0 (2)
C21—N1—N2	112.18 (17)	O3—C4—H4A	109.7
C21—N1—C1	127.50 (16)	C5—C4—H4A	109.7
N2—N1—C1	119.92 (16)	O3—C4—H4B	109.7
C23—N2—N1	104.46 (17)	C5—C4—H4B	109.7
N1—C1—C11	111.05 (15)	H4A—C4—H4B	108.2
N1—C1—C2	108.18 (15)	C21—C22—C23	105.97 (19)
C11—C1—C2	112.80 (16)	C21—C22—H22	127.0
N1—C1—H1	108.2	C23—C22—H22	127.0
C11—C1—H1	108.2	C4—C5—H5A	109.5
C2—C1—H1	108.2	C4—C5—H5B	109.5
C3—C2—C6	105.57 (15)	H5A—C5—H5B	109.5
C3—C2—C1	110.98 (16)	C4—C5—H5C	109.5
C6—C2—C1	110.86 (16)	H5A—C5—H5C	109.5
C3—C2—H2	109.8	H5B—C5—H5C	109.5
C6—C2—H2	109.8	C21—C25—H25A	109.5
C1—C2—H2	109.8	C21—C25—H25B	109.5
C12—C11—C16	118.10 (18)	H25A—C25—H25B	109.5
C12—C11—C1	120.23 (17)	C21—C25—H25C	109.5
C16—C11—C1	121.66 (17)	H25A—C25—H25C	109.5
O2—C3—O3	125.32 (19)	H25B—C25—H25C	109.5
O2—C3—C2	124.59 (18)	C8—C7—O5	108.6 (2)
O3—C3—C2	110.01 (16)	C8—C7—H7A	110.0
C13—C12—C11	121.13 (19)	O5—C7—H7A	110.0
C13—C12—H12	119.4	C8—C7—H7B	110.0
C11—C12—H12	119.4	O5—C7—H7B	110.0
N1—C21—C22	106.12 (18)	H7A—C7—H7B	108.3
N1—C21—C25	123.09 (19)	C23—C24—H24A	109.5
C22—C21—C25	130.8 (2)	C23—C24—H24B	109.5
C15—C16—C11	121.46 (19)	H24A—C24—H24B	109.5
C15—C16—H16	119.3	C23—C24—H24C	109.5
C11—C16—H16	119.3	H24A—C24—H24C	109.5
C12—C13—C14	120.21 (19)	H24B—C24—H24C	109.5
C12—C13—H13	119.9	O1—C17—H17A	109.5
C14—C13—H13	119.9	O1—C17—H17B	109.5
O1—C14—C15	124.7 (2)	H17A—C17—H17B	109.5
O1—C14—C13	115.74 (19)	O1—C17—H17C	109.5
C15—C14—C13	119.52 (19)	H17A—C17—H17C	109.5
C16—C15—C14	119.56 (19)	H17B—C17—H17C	109.5
C16—C15—H15	120.2	C7—C8—H8A	109.5
C14—C15—H15	120.2	C7—C8—H8B	109.5
O4—C6—O5	124.9 (2)	H8A—C8—H8B	109.5
O4—C6—C2	124.10 (19)	C7—C8—H8C	109.5
O5—C6—C2	110.92 (18)	H8A—C8—H8C	109.5
N2—C23—C22	111.27 (19)	H8B—C8—H8C	109.5

### Hydrogen-bond geometry (Å, °)

**Table:**

*D*—H···*A*	*D*—H	H···*A*	*D*···*A*	*D*—H···*A*
C12—H12···O2^i^	0.93	2.51	3.358 (3)	152

Symmetry codes: (i) −*x*+1, −*y*, −*z*+1.
